# Treatment potential of pathogen-reactive antibodies sequentially purified from pooled human immunoglobulin

**DOI:** 10.1186/s13104-019-4262-8

**Published:** 2019-04-15

**Authors:** Mark Reglinski, Shiranee Sriskandan

**Affiliations:** Faculty of Medicine, Imperial College London, Hammersmith Campus, Du Cane Road, London, W12 0NN UK

**Keywords:** IVIG, Pooled human immunoglobulin, Opsonophagocytic killing, *Staphylococcus aureus*, *Enterococcus*, Antimicrobial resistance

## Abstract

**Objective:**

Intravenous immune globulin (IVIG), pooled from human blood, is a polyspecific antibody preparation that inhibits the super-antigenic proteins associated with streptococcal and staphylococcal toxic shock, and the Shiga toxin. In addition to this toxin-neutralising activity, IVIG contains other pathogen-reactive antibodies that may confer additional therapeutic benefits. We sought to determine if pathogen-reactive antibodies that promote opsonophagocytosis of different organisms can be sequentially affinity-purified from one IVIG preparation.

**Results:**

Antibodies that recognise cell wall antigens of *Streptococcus pyogenes*, *Staphylococcus aureus*, and vancomycin-resistant enterococcus (VRE) were sequentially affinity-purified from a single preparation of commercial IVIG and opsonophagocytic activity was assessed using a flow cytometry assay of neutrophil uptake. Non-specific IgG-binding proteins were removed from the *S. aureus* preparations using an immobilised Fc fragment column, produced using IVIG cleaved with the Immunoglobulin G-degrading enzyme of *S. pyogenes* (IdeS). Affinity-purified anti-*S. aureus* and anti-VRE immunoglobulin promoted significantly higher levels of opsonophagocytic uptake by human neutrophils than IVIG when identical total antibody concentrations were compared, confirming activity previously shown for affinity-purified anti-*S. pyogenes* immunoglobulin. The opsonophagocytic activities of anti-*S. pyogenes*, anti-*S. aureus*, and anti-VRE antibodies that were sequentially purified from a single IVIG preparation were undiminished compared to antibodies purified from previously unused IVIG.

**Electronic supplementary material:**

The online version of this article (10.1186/s13104-019-4262-8) contains supplementary material, which is available to authorized users.

## Introduction

Intravenous immune globulin (IVIG) is a clinical antibody preparation purified from pooled human plasma obtained from at least 1000 donors [1]. Previously we demonstrated that immunoglobulins reactive against cell wall components of the human pathogen *Streptococcus pyogenes* can be purified from IVIG. In a murine model we used these immunoglobulins to reduce the severity and microbial burden of invasive *S. pyogenes* infection [2]. A small number of pre-clinical and in vitro studies have shown that IVIG has activity against other bacterial pathogens [3–5], including *Staphylococcus aureus* and *Enterococcus* spp., which have emerged as important agents of antimicrobial-resistant infections. The reported activity of polyspecific IVIG against *S. aureus* in vitro and in a rabbit pneumonia model [6, 7] and against *Enterococcus* spp. in an in vitro model of opsonic killing [4, 8] coupled with the antimicrobial efficacy of *S. pyogenes*-reactive “enhanced” (E)-IVIG in vivo [2] led us to investigate if antibody pools with enhanced opsonic activity against *S. aureus* and *Enterococcus* could be recovered from a commercial IVIG preparation. As the preparation of IVIG requires many blood donations and is costly, limiting its supply in some parts of the world, we also determined whether different pathogen-reactive antibody pools could be sequentially purified from a single vial of IVIG, maximising the potential yield of this approach.

## Main text

### Materials and methods

#### Bacterial strains and growth conditions

Five vancomycin-resistant enterococcal isolates (H1544-H1548) from routine rectal screening were cultured overnight in Todd-Hewitt broth at 37 °C + 5% CO_2_. *S. aureus* isolates were cultured overnight in brain–heart infusion at 37 °C with agitation at 225 rpm. For immunoglobulin-binding protein removal from *S. aureus,* pilot studies were conducted using clinical isolates HSS354-356. For E-IVIG preparation, five *S. aureus* CC8 lineage isolates were used: USA300, Newman, NCTC8325 [9], HHS-4 and HHS-5 [10]. Opsonophagocytosis assays were performed using Oregon-green 488 labelled methicillin-resistant *S. aureus* isolate USA300, *S. pyogenes emm*1 bacteraemia isolate H364 [2], and VRE isolate H1548.

#### Protein preparation and immobilization

To generate separate Fc and F(ab) fragments, IVIG consisting of ≥ 98% IgG (Privigen, CSL Behring) [11] was digested with recombinant Immunoglobulin G-degrading enzyme of *S. pyogenes* (IdeS) as previously described [12] and purified as outlined in Additional file 1: Methods. For resin immobilization, purified Fc fragments were dialysed into coupling buffer (0.1 M sodium bicarbonate, 0.5 M sodium chloride; pH 8.3) overnight at 4 °C. Coupling to cyanogen bromide activated agarose resin (CNBr) (Sigma Aldrich) was performed at a protein concentration of 1 mg/ml according to the manufacturer’s instructions.

Staphylococcal and enterococcal cell wall extracts (CWE) were prepared from five individual isolates, as previously described [2] using 1 mg/ml lysostaphin in place of lysozyme for *S. aureus*. To remove the IgG-binding proteins Sbi and protein A [13], staphylococcal CWEs were passed over the prepared Fc fragment column twice. The Fc column was stripped with 0.5 M NaOH and washed extensively with PBS between samples. To demonstrate adequate removal of IgG-binding proteins, 5 µl aliquots of *S. aureus* CWE from three clinical isolates were spotted onto a Hybond-LFP membrane (GE Healthcare) before and after IgG binding protein removal. Membranes were blocked with 5% (w/v) skim milk powered (Sigma Aldrich) in PBST and probed with 5 µg/ml of SEC purified Fc fragments (diluted in blocking buffer). Following three washes in PBST, membranes were incubated in a 1: 80,000 dilution of HRP-conjugated goat anti human IgG (Fc specific, Abcam). Bound antibodies were detected using ECL prime western blotting detection reagent (GE Healthcare) and exposure to chemiluminescent film (Amersham Hyperfilm ECL, GE Healthcare). In later experiments, IgG binding protein-depleted staphylococcal CWEs were separated by SDS-PAGE and transferred onto Hybond-LFP in duplicate. The resulting membrane was split, and probed with 5 µg/ml of IVIG, or 5 µg/ml of IdeS-cleaved IVIG (used in place of purified Fc fragments) as described above. Following secondary antibody incubation, the membrane was reassembled and developed as described above. For resin immobilisation, equal quantities of CWE from the five CC8 *S. aureus* or enterococcal strains selected for study was pooled and coupled to CNBr as outlined above.

#### Preparation of enhanced IVIG

Streptococcal, Staphylococcal and Enterococcal-reactive Enhanced (E)-IVIG was affinity-purified from previously unused IVIG using immobilised CWEs as described previously and in Additional file 1: Methods [2]. For sequential purification of pathogen-reactive E-IVIG preparations, IVIG was passed over an *S. pyogenes* CWE column prepared previously [2], followed by the *S. aureus* CWE column or vice versa. Twice depleted IVIG was subsequently passed over the Enterococcal CWE column to produce the third, pathogen-reactive antibody pool.

#### Neutrophil opsonophagocytosis assays

Neutrophil opsonophagocytosis assays were performed as previously described [12] using freshly isolated human neutrophils to demonstrate uptake of opsonised, Oregon green 488-labelled *S. aureus* USA300, *S. pyogenes* isolate H364, or VRE isolate H1548.

### Results

#### Removal of IgG binding proteins from *S. aureus* cell wall extracts

**Fig. 1:**
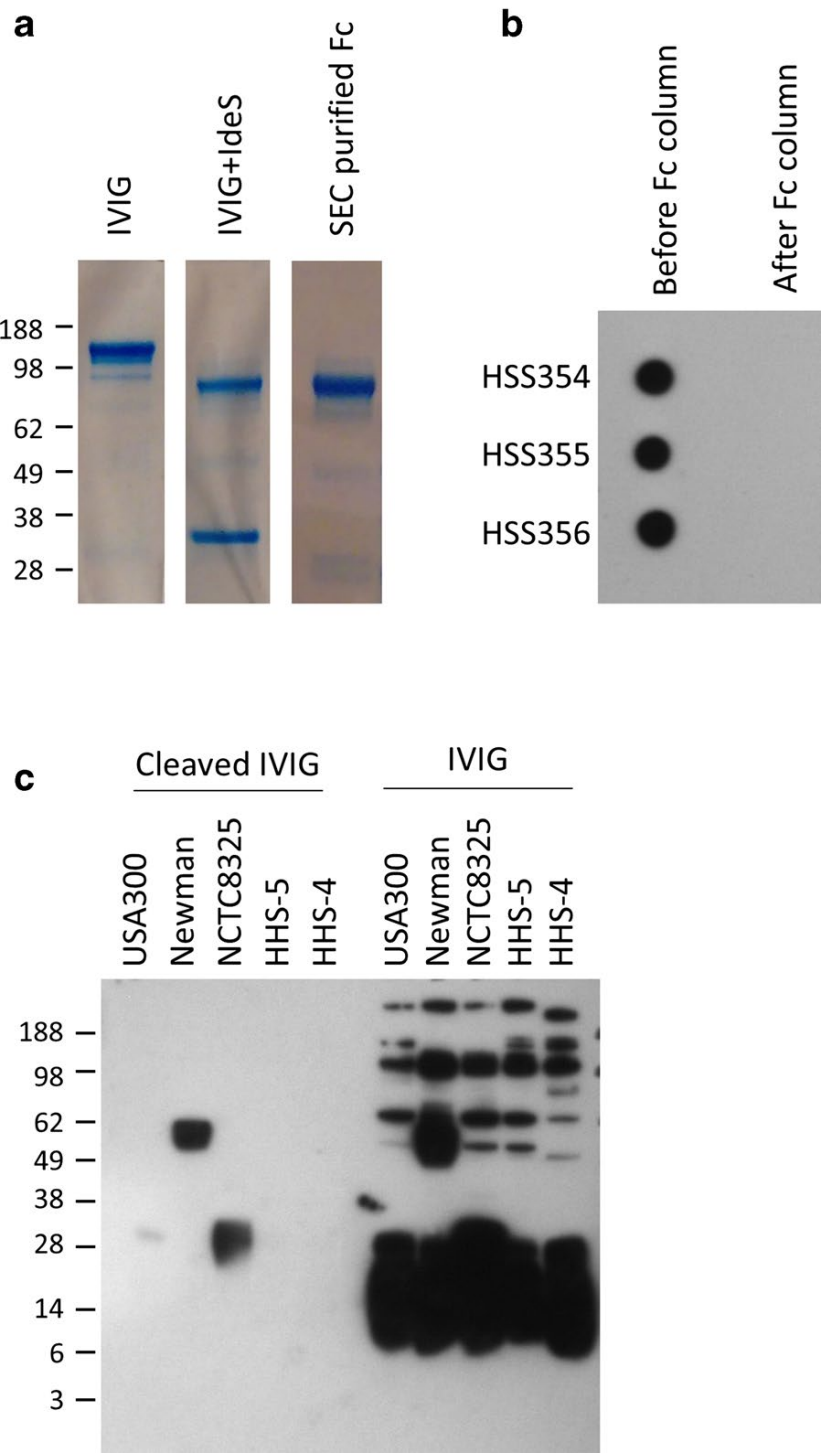
Removal of immunoglobulin binding proteins from *S. aureus* cell wall extracts by affinity chromatography. **a** Intravenous immune globulin (IVIG) was digested with recombinant IdeS and Fc fragments were purified by size exclusion chromatography (SEC) prior to immobilisation onto cyanogen bromide activated agarose. **b** 5 µl aliquots of *S. aureus* CWE from three clinical isolates were spotted onto a Hybond-LFP membrane before and after protein A removal and probed with 5 µg/ml of SEC purified Fc fragments. **c** 5 µg of protein A-depleted CWE from five *S. aureus* isolates (CC8) were visualised by immunoblotting using 5 µg/ml of IdeS-cleaved IVIG (left) or IVIG (right) as a primary antibody. For **b**, **c**, bound primary antibodies were detected by exposure to chemiluminescent film using a 1: 80,000 dilution of Fc specific goat anti-human IgG (HRP-conjugated) and ECL prime

We hypothesised that opsonic antibodies could be purified from IVIG by affinity chromatography using cell wall antigens from different bacterial pathogens. To prevent non-specific antibody purification, the IgG binding proteins Sbi and protein A were removed from the *S. aureus* CWEs prior to affinity resin preparation using an immobilised Fc column produced from IdeS-cleaved IVIG (Fig. 1a). Passing CWE from three clinical *S. aureus* isolates over the Fc column reduced levels of non-specific IgG binding (visualised using purified Fc fragments) below the limit of detection of the ECL prime reagent, indicating that the majority of non-specific immunoglobulin-binding proteins had been removed from the samples (Fig. 1b).

To confirm the presence of conserved, IVIG-reactive antigens across the clinically important CC8 *S. aureus* linage, CWEs were prepared from five CC8 isolates, and IgG-binding proteins were depleted as outlined above. IVIG-reactive proteins were visualised by immunoblotting in duplicate, using either intact IVIG or IdeS-cleaved IVIG as primary antibodies, and Fc-specific secondary antibody. Immunoblot analysis using intact IVIG revealed a common banding pattern, suggesting that many recognised antigens may be conserved among CC8 isolates (Fig. 1c). IdeS-cleaved IVIG was used to reveal any residual proteins that bound non-specifically via the Fc domain, and confirmed that IgG binding protein removal had largely been successful in these samples. Carryover of IgG-binding proteins in the Newman and *S. aureus* 8325 samples was apparent, consistent with reported high levels of Sbi production by *S. aureus* Newman [13]. Accordingly, these samples were re-applied to the Fc column prior to affinity resin preparation.

#### Purification of opsonic pathogen-reactive immunoglobulin from IVIG using *S. aureus* and VRE cell wall extracts

**Fig. 2:**
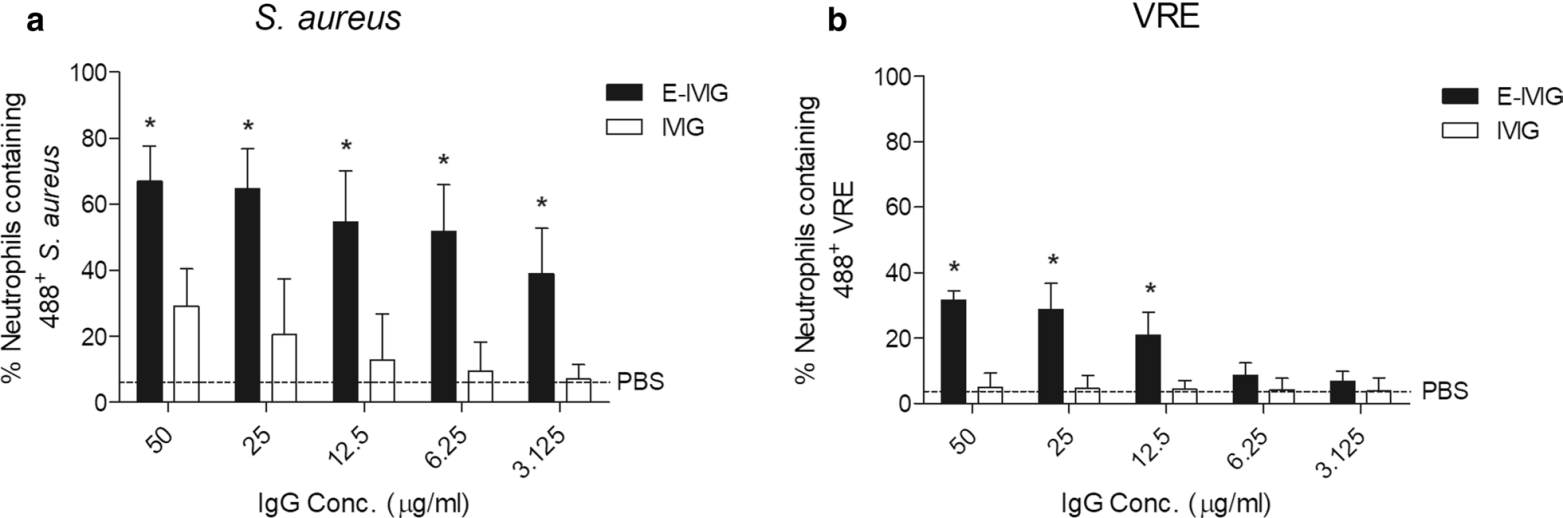
E-IVIG pools can promote opsonophagocytic uptake of *S. aureus* and VRE. *S. aureus* E-IVIG (**a**) promotes uptake of USA300, and VRE E-IVIG (**b**) promotes uptake of clinical VRE isolate H1548 (both E-IVIGs, black bars) compared with commercial IVIG (white bars), used at equal concentrations. Neutrophil uptake of bacteria in the absence of any IVIG is shown by the horizontal dotted line (PBS only). Results from three different neutrophil donors are displayed as the percentage of neutrophils containing Oregon green 488-X SE labelled bacteria (Mean ± SD). *p < 0.05, two-tailed Student’s *t* test

To determine if functional anti-staphylococcal immunoglobulin could be purified from commercially available IVIG using immobilised *S. aureus* CWE, an affinity purification column was prepared using the IgG binding protein-depleted CC8 CWEs. *S. aureus*-reactive E-IVIG was affinity-purified as outlined in “Materials and methods” section and the ability of the resulting eluted preparation to promote human neutrophil uptake of USA300 was compared to commercial IVIG. Following a 30 min co-incubation, the percentage of neutrophils containing labelled *S. aureus* USA300 was significantly higher in the presence of affinity-purified *S. aureus* E-IVIG compared to commercial IVIG at all concentrations examined (Fig. 2a).

Application of IVIG to an affinity-purification column prepared using CWE from five isolates of vancomycin-resistant enterococcus resulted in the generation of an E-IVIG preparation with enhanced anti-enterococcal activity in the same neutrophil assay, using one of the VRE clinical isolates as the target organism (Fig. 2b). Neutrophil uptake of VRE was however lower than observed for USA300, possibly reflecting the presence of fewer antigenic epitopes on the enterococcal cell surface. Together with previous data showing enhanced uptake of *S. pyogenes* with E-IVIG and other reports [2, 4], these data confirm that commercial IVIG contains opsonic antibodies that recognise *S. pyogenes*, *S. aureus* and *Enterococcus* spp., and that such antibodies can be purified and concentrated from commercial IVIG.

#### Pathogen-reactive antibodies can be sequentially purified from a single preparation of IVIG

**Fig. 3:**
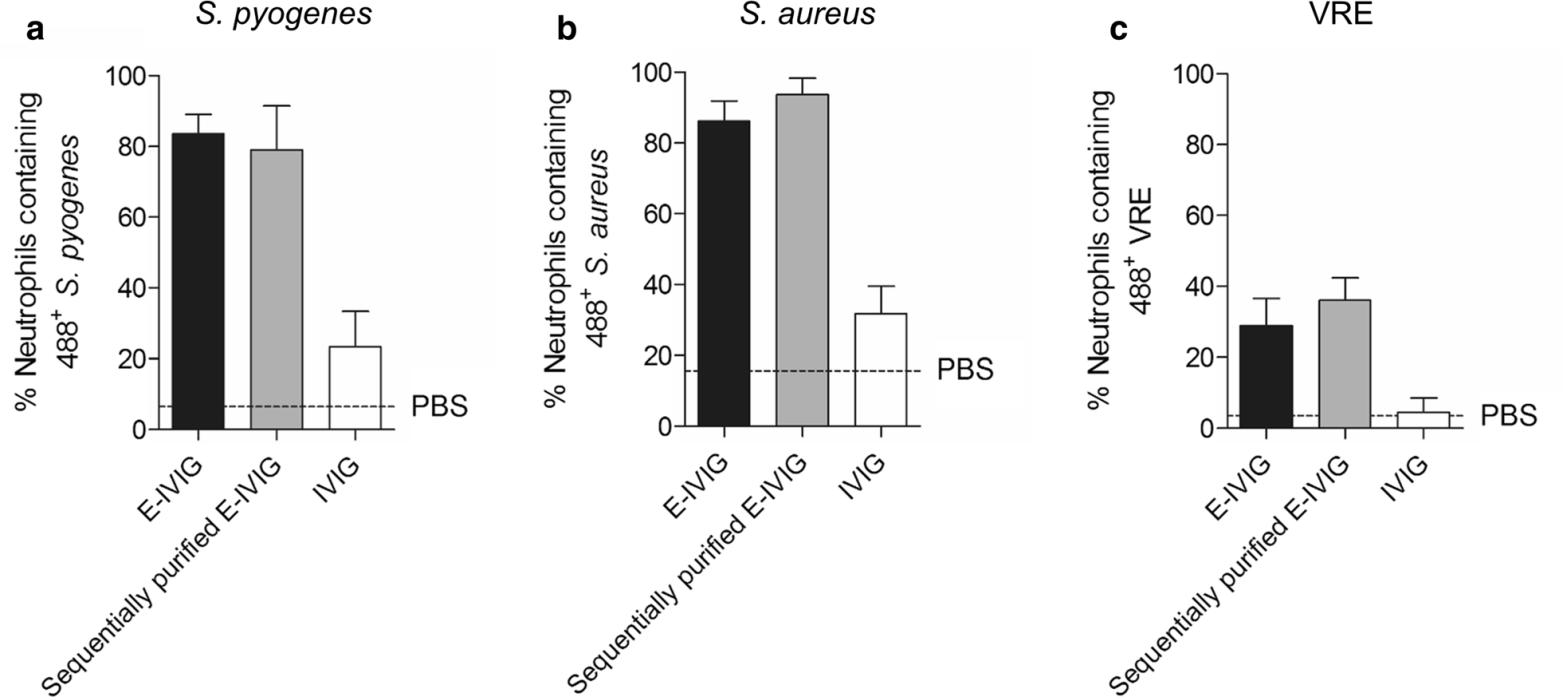
Pathogen-reactive E-IVIG can be sequentially purified from a single vial of IVIG without appreciable loss in opsonophagocytic activity. Black bars show activity of E-IVIG (primary purification from IVIG); grey bars show activity of sequentially-purified E-IVIG, (secondarily-purified from flow through fraction of an earlier, primary E-IVIG purification); white bars show activity of commercial IVIG. All immunoglobulin preparations were used at 25 µg/ml. **a** Anti *S. pyogenes* E-IVIG activity against H364. Sequentially purified E-IVIG was prepared from flow-through of primary *S. aureus* E-IVIG purification **b** Anti *S. aureus* E-IVIG activity against USA300. Sequentially purified E-IVIG was prepared from flow-through of primary *S. pyogenes* E-IVIG purification. **c** Anti-VRE E-IVIG activity against H1548. Sequentially purified E-IVIG was prepared from flow through obtained following purification of *S. pyogenes* E-IVIG and then *S. aureus* E-IVIG. Neutrophil uptake of bacteria in the absence of any IVIG is shown by the horizontal dotted line (PBS only). Results from three different neutrophil donors are displayed as the percentage of neutrophils containing Oregon green 488-X SE labelled bacteria (Mean ± SD)

To determine if pathogen-reactive antibodies can be consecutively purified from a single vial of IVIG, sequentially purified E-IVIG reactive against *S. pyogenes*, *S. aureus* and VRE was prepared as outlined in “Materials and methods” section using the flow through from a previous E-IVIG purification. These preparations were then assessed for opsonic activity. The level of neutrophil uptake promoted by the sequentially purified E-IVIG pools did not differ appreciably from the levels promoted by cognate E-IVIG preparations from previously unused IVIG at a fixed concentration of 25 µg/ml (Fig. 3). These data indicate that it is possible to sequentially purify at least three pools of pathogen-reactive antibodies from the same preparation of IVIG without a detectable reduction in opsonophagocytic activity, and without waste.

### Discussion

Intravenous immune globulin (IVIG) is a clinical blood product that is predicted to have activity against a variety of different bacterial and viral pathogens based on existing experimental, but not clinical, data. However, IVIG has not shown clear success in clinical sepsis trials [14]; perhaps in part because the concentration of pathogen-reactive antibody in IVIG is not sufficient to provide passive protection. Previously [2], and in this report, we have demonstrated that pools of *S. pyogenes*, *S. aureus* and Enterococcal-reactive antibodies with enhanced opsonic activity can be purified from commercial IVIG. In addition, our data indicate that such pools can be purified sequentially without an appreciable loss in opsonophagocytic activity; suggesting that further studies into the sequential purification of multiple, pathogen-reactive E-IVIG preparations from commercial IVIG are warranted.

In this report we did not investigate whether the order in which the immunoglobulin pools were purified impacted upon the functionality of the resulting E-IVIG preparations. It is conceivable that cross-reactive antibodies that recognise all three pathogens are present within the starting IVIG preparation. In our study, no difference in opsonic activity was detected between sequentially purified E-IVIG and E-IVIG prepared from previously unused IVIG against the three pathogens studied however, reductions in potency may become apparent if additional pathogens are included, and the number of sequential purifications undertaken is further increased.

Passive immunisation has previously been investigated as a treatment for invasive *S. aureus* infection, with the most promising antibody preparation (Altastaph) reaching phase two clinical trials prior to cessation of development [15, 16]. Altastaph is a polyspecific IgG preparation purified from patients vaccinated with recombinant conjugated preparations of *S. aureus* type 5 and type 8 capsular polysaccharide [15, 17]. Protection is therefore based on the presence anti-CPS antibodies; however, anti-type 5 CPS concentrations were estimated at 4% of the total administered IgG [17] indicating that the majority of immunoglobulin was not specific for *S. aureus*. In contrast, the affinity purification process applied herein dramatically increases the concentration of *S. aureus* or VRE-reactive immunoglobulins present within the reagent, which may improve the efficacy of passive immunisations markedly. Demonstration that such reagents could be useful in human disease will require additional experimental work. For example, it is widely recognised that neutrophil uptake assays and murine studies alone are not necessarily predictive of *S. aureus* clearance in human disease [18]. Nonetheless, as the pipeline of antimicrobial reagents slows down, the use of adjunctive immunological reagents that collaborate with the immune system represents a potentially useful approach to treatment of multidrug-resistant pathogens. As such, the use of affinity-purified E-IVIG is an approach that should be explored further.

## Limitations

- Neutrophil uptake was assessed using a flow cytometry-based assay which does not allow the viability of the associated bacteria to be assessed. The bactericidal activity of the E-IVIG preparations, either in vivo or in an opsonophagocytic killing assay, remains to be determined.
- Bacterial uptake has only been shown for a single isolate of *S. aureus* and VRE, although the ability of E-IVIG to promote uptake of multiple *S. pyogenes* isolates has previously been described [2].
- A single brand of IVIG was used, and we cannot extrapolate our findings to other commercial preparations without testing, noting that population exposure to certain pathogens may differ. *S. pyogenes* E-IVIG can however be purified from three different commercial IVIG preparations, suggesting that, for common pathogens, the ability to purify pathogen-reactive antibody pools is unlikely to be limited to specific IVIG preparations [2].

## Additional file

**Additional file 1.** Contains details of Fc fragment and E-IVIG purification protocols.

## Abbreviations

IVIG: intravenous immune globulin
VRE: vancomycin-resistant enterococcus
IdeS: immunoglobulin G-degrading enzyme of *S. pyogenes*
CNBr: cyanogen bromide activated agarose resin
CWE: cell wall extracts
E-IVIG: enhanced intravenous immune globulin

## References

[1] Seite JF, Shoenfeld Y, Youinou P, Hillion S (2008). What is the contents of the magic draft IVIg?. Autoimmun Rev.

[2] Reglinski M, Gierula M, Lynskey NN, Edwards RJ, Sriskandan S (2015). Identification of the *Streptococcus pyogenes* surface antigens recognised by pooled human immunoglobulin. Sci Rep.

[3] Mikolajczyk MG, Concepcion NF, Wang T, Frazier D, Golding B, Frasch CE (2004). Characterization of antibodies to capsular polysaccharide antigens of *Haemophilus influenzae* type b and *Streptococcus pneumoniae* in human immune globulin intravenous preparations. Clin Diagn Lab Immunol.

[4] Rossmann FS, Kropec A, Laverde D, Saaverda FR, Wobser D, Huebner J (2015). In vitro and in vivo activity of hyperimmune globulin preparations against multiresistant nosocomial pathogens. Infection.

[5] Ashkenazi S, Cleary TG, Lopez E, Pickering LK (1988). Anticytotoxin-neutralizing antibodies in immune globulin preparations: potential use in hemolytic-uremic syndrome. J Pediatr.

[6] Diep BA, Le VT, Badiou C, Le HN, Pinheiro MG, Duong AH (2016). IVIG-mediated protection against necrotizing pneumonia caused by MRSA. Sci Transl Med.

[7] Farag N, Mahran L, Abou-Aisha K, El-Azizi M (2013). Assessment of the efficacy of polyclonal intravenous immunoglobulin G (IVIG) against the infectivity of clinical isolates of methicillin-resistant *Staphylococcus aureus* (MRSA) in vitro and in vivo. Eur J Clin Microbiol Infect Dis.

[8] Sallam MM, Abou-Aisha K, El-Azizi M (2016). A novel combination approach of human polyclonal IVIG and antibiotics against multidrug-resistant Gram-positive bacteria. Infect Drug Resist.

[9] Enright MC, Robinson DA, Randle G, Feil EJ, Grundmann H, Spratt BG (2002). The evolutionary history of methicillin-resistant *Staphylococcus aureus* (MRSA). Proc Natl Acad Sci U S A.

[10] Smith DS, Siggins MK, Gierula M, Pichon B, Turner CE, Lynskey NN (2016). Identification of commonly expressed exoproteins and proteolytic cleavage events by proteomic mining of clinically relevant UK isolates of *Staphylococcus aureus*. Microb Genom.

[11] Privigen Prescribing Information. https://www.privigen.com/prescribing-information. Accessed 23 Mar 2019.

[12] Reglinski M, Lynskey NN, Sriskandan S (2016). Modification of the classical Lancefield assay of group A streptococcal killing to reduce inter-donor variation. J Microbiol Methods.

[13] Zhang L, Jacobsson K, Vasi J, Lindberg M, Frykberg L (1998). A second IgG-binding protein in *Staphylococcus aureus*. Microbiology.

[14] Alejandria M, Lansang M, Dans L, Mantaring J (2013). Intravenous immunoglobulin for treating sepsis, severe sepsis and septic shock. Cochrane Database Syst Rev.

[15] Rupp ME, Holley HP, Lutz J, Dicpinigaitis PV, Woods CW, Levine DP (2007). Phase II, randomized, multicenter, double-blind, placebo-controlled trial of a polyclonal anti-*Staphylococcus aureus* capsular polysaccharide immune globulin in treatment of *Staphylococcus aureus* bacteremia. Antimicrob Agents Chemother.

[16] Garcia-Lara J, Foster SJ (2009). Anti-*Staphylococcus aureus* immunotherapy: current status and prospects. Curr Opin Pharmacol.

[17] Fattom AI, Sarwar J, Ortiz A, Naso R (1996). A *Staphylococcus aureus* capsular polysaccharide (CP) vaccine and CP-specific antibodies protect mice against bacterial challenge. Infect Immun.

[18] Fowler VG, Proctor RA (2014). Where does a *Staphylococcus aureus* vaccine stand?. Clin Microbiol Infect.

