# Supplementary material for: Treatment potential of pathogen-reactive antibodies sequentially purified from pooled human immunoglobulin
Source: BMC Res Notes. 2019 Apr 15;12:228. doi: 10.1186/s13104-019-4262-8 (PMC6466806; doi:10.1186/s13104-019-4262-8)
Supplement: Supplementary file 1 — Additional file 1. Contains details of Fc fragment and E-IVIG purification protocols. [file 13104_2019_4262_MOESM1_ESM.docx]

**Additional file 1:Methods**

**Purification of Fc fragments**

IdeS-cleaved Fc fragments were separated by size exclusion chromatography (SEC) using a 50 cm Econo-Column (Bio-Rad) packed with Sephadex G-100 (Pharmacia). Fractions containing purified Fc fragments were identified by SDS-PAGE using the Novex NuPAGE SDS-PAGE Gel System (Thermo Fisher) and InstantBlue staining (Expedeon) according to the manufacturer’s instructions. Fc fragment-containing fractions were pooled, concentrated using 10,000 MWCO filter concentrators (Amicon Ultra, Millipore) and protein concentrations were determined using the Pierce Coomassie Plus (Bradford) Assay Kit (Thermo Fisher).

**Preparation of Enhanced IVIG**

Resin immobilised CWEs were incubated with 5 ml of 5 mg/ml IVIG for 2 h at RT and washed extensively with PBS. Bound antibodies were eluted into 1 ml aliquots of 1 M acetic acid and immediately neutralised using an equal volume of 3 M Tris-HCl (pH 8.8). Eluted fractions (containing the E-IVIG) were pooled, dialysed into PBS and protein (immunoglobulin) concentrations were determined by Bradford assay as outlined above.
